# Automated detection of stale beef from electronic nose data

**DOI:** 10.1002/fsn3.3910

**Published:** 2024-10-28

**Authors:** Wenshen Jia, Haolin Lv, Yang Liu, Wei Zhou, Yingdong Qin, Jie Ma

**Affiliations:** ^1^ Institute of Quality Standard and Testing Technology Beijing Academy of Agriculture and Forestry Sciences Beijing China; ^2^ Department of Risk Assessment Lab for Agro‐Products (Beijing) Ministry of Agriculture and Rural Affairs Beijing China; ^3^ Key Laboratory of Urban Agriculture (North China) Ministry of Agriculture and Rural Affairs Beijing China; ^4^ Lu'an Branch Anhui Institute of Innovation for Industrial Technology Lu'an China; ^5^ College of Computer and Information China Three Gorges University Yichang China; ^6^ Mechanical Electrical Engineering School, Beijing Information Science and Technology University Beijing China; ^7^ Food Inspection and Research Institute Hebei Food Safety Key Laboratory Shijiazhuang China; ^8^ College of Intelligent Science and Engineering Beijing University of Agriculture Beijing China

**Keywords:** back propagation neural network, confusion matrix, electronic nose, k‐nearest neighbor, linear discriminant analysis, machine learning, principal component analysis, stale beef, support vector machine

## Abstract

Accurate detection of stale beef on the market is important for protecting the legitimate rights and interests of consumers. To this end, we combined electronic nose measurements with machine learning technology to classify beef samples. We used an electronic nose to collect information about the odor characteristics of different beef samples and used linear discriminant analysis to reduce data dimensionality. We then classified samples using the following algorithms: extreme gradient boosting, logistic regression, K‐nearest neighbor, random forest, support vector machine, and neural networks for pattern recognition. We assessed model performance using a 10‐fold cross‐validation technique. All these methods reached an accuracy of 95% or above, with *F*1 scores and AUC values above 0.96. The support vector machine algorithm outperformed all other models, achieving perfect recognition with 100% accuracy and *F*1/AUC scores of 1.0. Our study demonstrates that electronic nose data combined with support vector machine can be used to successfully discriminate between stale and fresh beef, paving the way for novel research directions in the detection of stale beef.

## Graphical abstract

The study explores beef storage's effect on quality, emphasizing source comparisons to safeguard consumers from spoiled beef risks. E‐nose data and ML enable precise stale beef classification. Study uses diverse ML methods incl. feature extraction, ensemble learning, PCA. E‐nose + SVM achieves stable 100% recognition, ensuring reliable spoiled beef detection.

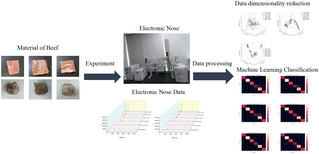

## 1 INTRODUCTION

Beef is rich in many essential nutrients, such as zinc and magnesium (Djinovic‐Stojanovic et al., 2017), which can help the body synthesize protein, promote muscle growth, and accelerate the rate of insulin production in the body (Duan et al., 2015). Beef also contains vitamin B6 (Meyer et al., 1969), which can enhance the immune system. Although beef is low in fat, it is rich in linoleic acid (Daley et al., 2010). Eating beef regularly helps restore muscle damage caused by strenuous exercise, and therefore helps athletes maintain good health and muscle mass. In addition, beef is rich in nutrients such as iron and alanine, which play an important role in the growth and development of the human body. Although beef is typically more expensive than pork and chicken, steady improvement in living standards has led to a substantial increase in the demand for beef and beef products. The freshness of beef directly affects the quality, taste, and nutritional value of beef. When cows are slaughtered, ribonucleotides in muscles are broken down into free ribose, hypoxanthine, and phosphate. Related metabolites from proteolysis and glycogen depletion lead to an increase in free amino acids, and these reactions directly affect the flavor and texture of beef when cooked (Koutsidis et al., 2008). At present, most countries in the world store a certain amount of beef as national strategic storage material. Some unscrupulous businessmen smuggle strategic reserve beef that has expired (after 2 years of storage) at a low price, and then introduce it into our market. Once circulated, expired beef poses a serious threat to the personal health and safety of consumers. To protect consumers and combat illegal smuggling and selling of stale beef, a method is urgently needed to detect and identify this product.

Animal noses (including ours) can theoretically identify hundreds of thousands of different odors. Electronic noses (also called artificial noses) have therefore been inspired by the sense of smell of humans and animals. These devices consist of a multi‐sensor array that can detect a variety of chemical components in the air (Karakaya et al., 2019; Wasilewski et al., 2017). Recent years have seen rapid development of sensor technology and artificial intelligence technology. When combined with artificial intelligence technology, electronic noses have played an important role in the qualitative and quantitative analysis of food quality. Tan and Xu (2020) pointed out that electronic nose technology combined with pattern recognition algorithms represent powerful analytical tools in the field of food inspection, carrying the advantages of low cost, rapid detection, and applicability to both online and offline detection. At present, the methods used for food quality detection include GC–MS (Gas chromatography mass spectrometry), SHS‐GC‐IMS (Static Headspace‐Gas Chromatography‐Ion Mobility Spectroscopy), PCR, and DNA technology. In addition to requiring expensive equipment, these detection methods must be operated by specialized personnel in dedicated laboratories. Compared with these methods, electronic noses present the advantages of simple operation, convenience, and non‐destructive testing. Džermeikaitė et al. (2023) discuss the many challenges involved in the application of modern technologies to dairy farms for objectively evaluating sensor methods and systems. With the availability of sensors and high‐precision technology for real‐time monitoring of cattle, it becomes important to assess the long‐term impact of these technologies on the overall sustainability of farms. This assessment includes evaluating their contribution to productivity, health monitoring, welfare evaluation, and environmental effects. Ouyang et al. (2019) employed a portable electronic tongue integrated with chemometric algorithms to detect the overall content of theaflavins in black tea. The authors utilized the synergy interval partial least squares with competitive adaptive reweighted sampling (Si‐CARS‐PLS) method, which yielded a prediction performance of Rp = 0.8302 and RMSEP = 0.257. Qin and Jia (2023) conducted a study investigating the release of volatile ammonia and sulfides during rabbit breeding using electronic components, including gas sensors. They further developed a specialized electronic nose by combining more advanced gas sensor combinations and incorporating machine learning techniques. This electronic nose was specifically designed for detecting minor damage in strawberries, and achieved an accuracy rate of over 80% (Qin et al., 2023). Xiao et al. (2014) used an electronic nose to measure the total viable count (TVC) index and total volatile basic nitrogen (TVB‐N) index of beef tenderloin samples over a period of 10 days, and then established a model for classifying this product. Their experimental results show that their method is successful at predicting the freshness of beef tenderloin samples. Hong et al. (2012) used an electronic nose to collect gas information from beef loin every 2 days for a total of seven times, and used a generalized regression neural network model to predict the storage time of beef loin. With a standard error on the predicted result of 1.36 days, their results show that electronic noses present great potential for predicting the freshness of beef.

Deep learning is at the forefront of machine learning research and includes advanced techniques such as recurrent neural networks (RNN) (Onan, 2020) and graph neural networks (GNN) (Onan, 2023a). The field has seen significant advances with the emergence of heuristic algorithms, ushering in a new era of machine learning. Approaches such as ant colony optimization (ACO) (Onan, 2023b) and genetic algorithms (GA) (Onan & Korukoğlu, 2017) have given traditional machine learning methods a more human‐like capability. Adulteration in beef also poses a serious threat to beef quality. Wakhid et al. (2022) used an electronic nose to detect adulteration of different beef samples. In their study, adulterated beef was effectively discriminated through feature extraction and ensemble learning on the original sample data, with a top accuracy of 95.71%. Valentin et al. (2021) used natural language processing technology to analyze the relationship between animal diseases and popular diseases in the news, and showed that this approach was more successful at predicting the national geographical location of related diseases. Huang and Gu (2022) used an electronic nose combined with a one‐dimensional convolutional neural network random forest regressor (1‐DCNN‐RFR) for detecting adulteration in meat. These authors used 1‐DCNN as the backbone of their model for extracting feature vectors from electronic nose data, and then introduced RFR as regressor on the model. Their experiments have shown that this method is effective at detecting and quantifying meat adulteration. Lu et al. (2021) used an electronic tongue consisting of six metal electrodes combined with machine learning to detect adulterated beef. The results indicated that ELM‐derived recognition models achieved high accuracy, with recognition rates exceeding 90% for independent samples from various meat groups. Bakhshipour (2023) combined electronic nose with spectroscopy and used SVM, PLSDA, SVR methods for data processing to track and detect the ripening process of kiwifruit.

PCA‐related dimensionality reduction methods have played a crucial role in research involving electronic noses. Han et al. (2020) used an electronic nose and an electronic tongue to collect feature information from red wine samples. Their identification model derived from PCA combined with extreme learning was able to correctly identify origin and brand of red wine. Chen et al. (2021) used an electronic nose to examine the exhaled breath of patients, and implemented KPCA combined with the extreme gradient boosting method to successfully identify and discriminate lung cancer, smoking‐related conditions, and other respiratory diseases. Tăuţan et al. (2021) used PCA to reduce data dimensionality before building a model for automatic detection of sleep stages. Their experimental results show that PCA can reduce computational load while leaving performance unaffected, and in most cases actually improving model accuracy.

In recent years, stale beef has seriously threatened the personal health and safety of consumers. At present, most research focuses on the effect of short‐term storage (<1 month) on beef quality. There are only a few studies on the identification and detection of stale beef stored for more than 1 year. To increase our knowledge in the area of long‐term storage, our study includes the following elements: (1) adoption of an electronic nose to collect characteristic information from fresh beef and stale beef; (2) preprocessing of collected feature information; (3) integration of machine learning tools to establish a suitable pattern recognition model for detecting stale beef.

## 2 MATERIALS AND METHODS

### 2.1 Materials

**FIGURE 1:**
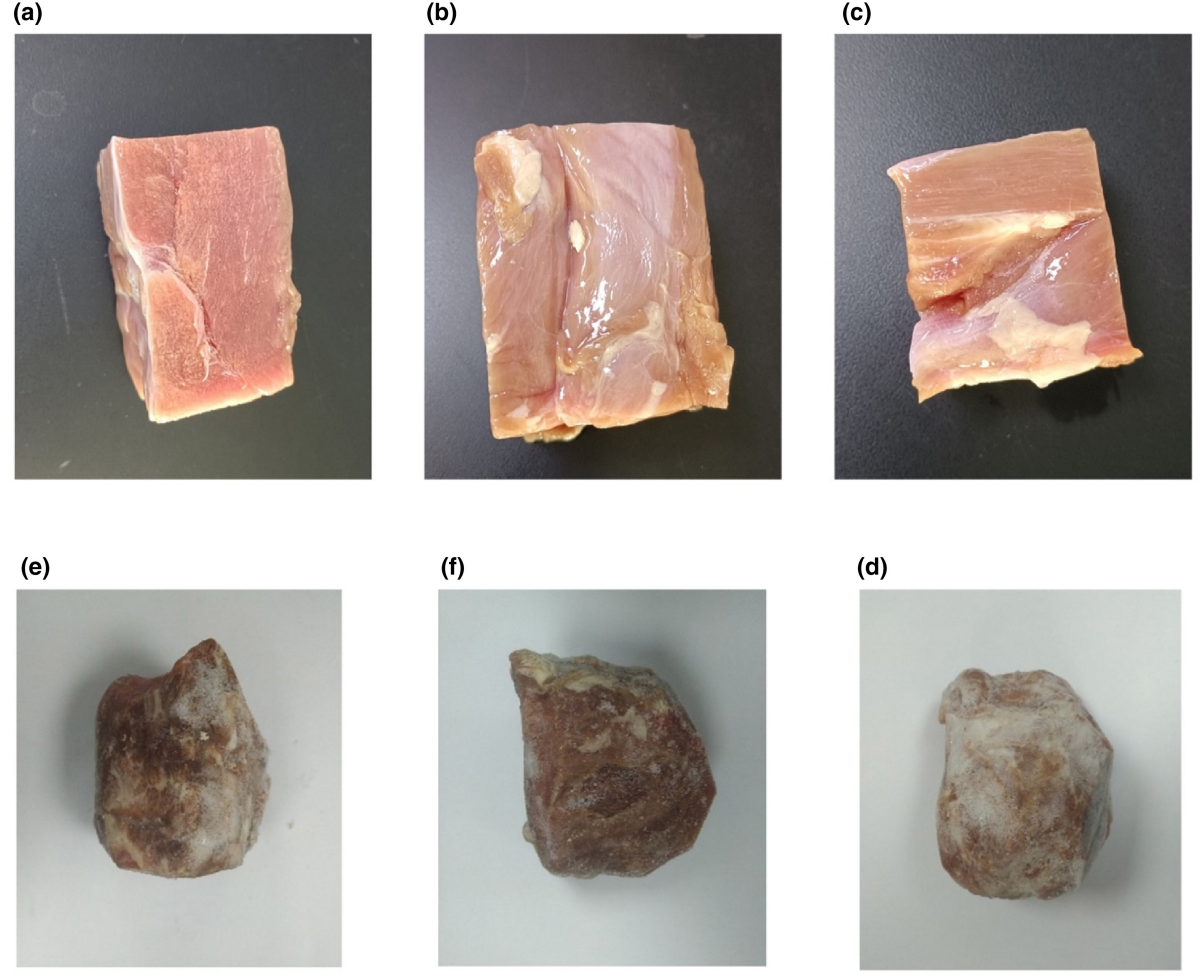
Fresh and stale beef samples from different origins. Fresh beef from Brazil (a), Canada (b), and Horqin (c). Stale beef from Brazil (d), Canada (e), and Horqin (f).

The 35 beef samples used in this experiment were from Brazil (8 samples), Canada (17 samples), and Horqin (10 samples; see Figure 1 for some examples).

Beef samples were collected every 20 min for a total of three times. We collected 105 groups of fresh beef data. The stale beef used in the experiment comes from fresh beef stored in the −20°C refrigerator for 2 years. Stale beef data were collected in parallel during three sessions with 105 groups. Before the experiment, the tested beef sample was taken out of the refrigerator, placed on a clean workbench. After dividing the sample into portions weighing 2.0 g (as measured using an electronic analytical balance), They were stored into vials and sealed with a screw cap for use. The prepared samples were kept at room temperature for 30 min to be classified, with each sample being sampled three times.

**FIGURE 2:**
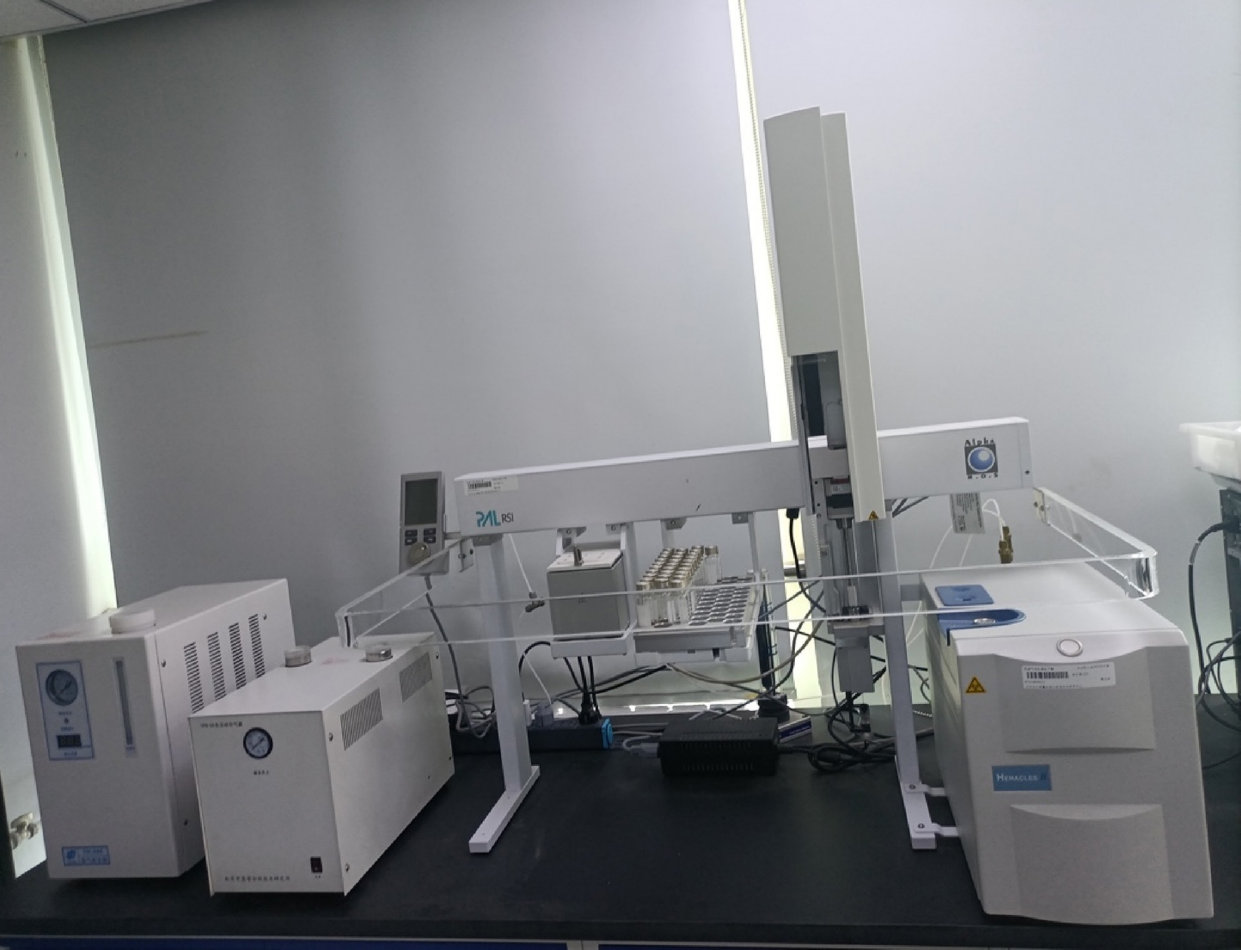
Heracles II ultra‐fast gas chromatography electronic nose.

**FIGURE 3:**
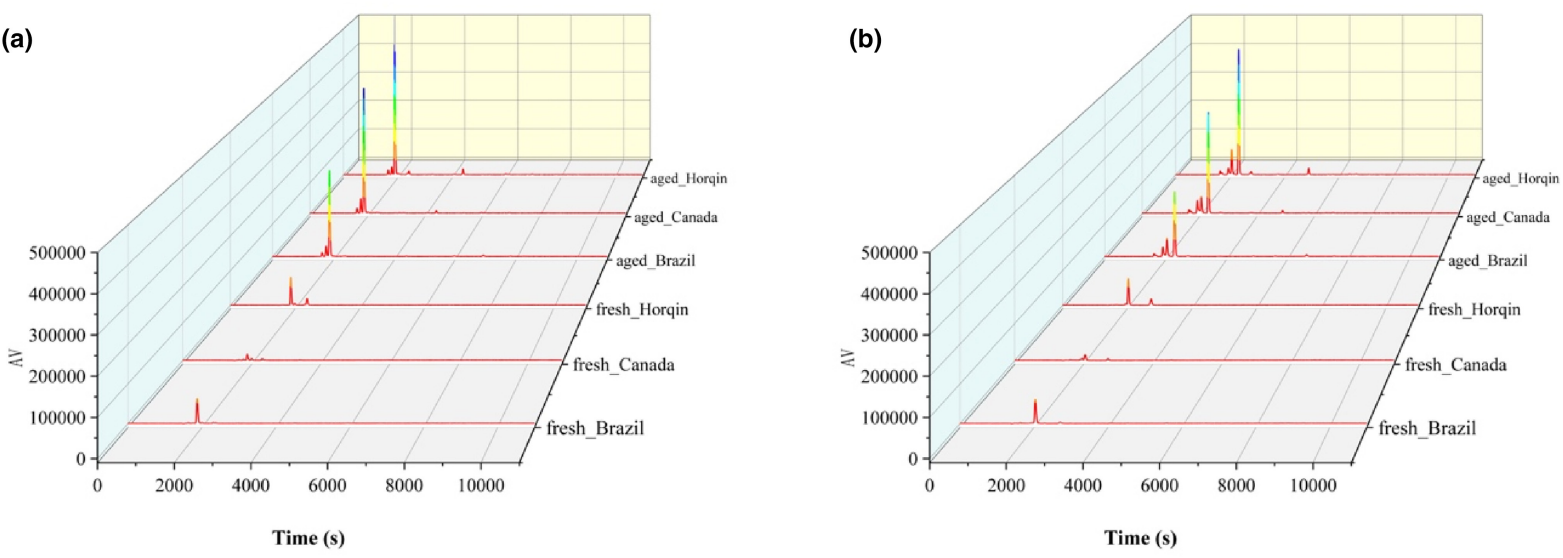
Chromatograms of fresh and stale beef from different origins. (a) Non‐polar column chromatograms of fresh and stale beef from different origins; (b) weak polar column chromatograms of fresh and stale beef from different origins.

To ensure validity of the resulting data, we set identical parameters for the electronic nose when collecting data from fresh beef and stale beef. We used a Heracles II ultra‐fast gas chromatography electronic nose (Alpha MOS, France; see Figure 2), a computer with AlphaSoft software, a TH‐300 hydrogen generator, and an SPB‐3S automatic air source (Beijing Zhonghe Institute of Analytical Technology). All experimental parameters are detailed in Table 1. We established the experimental method and experimental sequence in AlphaSoft software, put the sample vials into the tray in sequence, and then ran the entire electronic nose system to collect data. Our electronic nose has two chromatographic columns with different polarities: MXT‐5 (non‐polar chromatographic column) and MXT‐1701 (weakly polar chromatographic column). The two chromatographic columns operate simultaneously during data collection. We recorded peak time and peak value of the sample in the characteristic chromatogram. During the experiment, data collection time was 110 s for each sample, with sampling frequency of 100 Hz. Therefore, the characteristic information from each sample consists of 22,000 data points. Chromatograms of fresh and stale beef are shown in Figure 3.

**TABLE 1:** Experimental parameters used for both stale and fresh beef.

Instrument		Parameter	Set value	Instrument	Parameter	Set value
Analyzer	Injector	Injection volume	5000 μL	Trap	Capture temperature	40°C
		Injection speed	500 μL/s		Pre‐column pressure	57 kPa
		Inlet temperature	200°C		Shunt speed	10 mL/min
		Inlet pressure	10 kPa		Constant current speed	1.0 mL/min
		Export speed	30 mL/min		Capture time	30 s
		Injection time	15 s		Preheating time	35 s
	Valve	Valve temperature	250°C		Transmission time	10 s
					Original pressure	80 kPa
	Column oven	Initial furnace temperature	50°C		Final pressure	180 kPa
		Initial temperature line	2		Rate	1.67 kPa/s
		Data collection time	110 s	FID	Detector temperature	260°C
		Data collection cycle	0.01 s		Gain	12
					Offset	1000
Autosampler	Heater	Incubation period	25 min	Heating shaker	Stirring speed	500 rpm
		Incubation temperature	60°C		Start stirring	5 s
Autosampler	Syringe	Cleaning time	90 s	Heating shaker	Stop stirring	2 s
		Syringe temperature	50°C			
		Filling speed	500 μL/s			

### 2.2 Data preprocessing

During data collection, the electronic nose is affected by some inevitable factors, such as the nature of the surrounding environment and equipment performance. These factors will cause the electronic nose to generate abnormal data, which in turn may lead to inconsistent or even opposite results. In order to ensure reliability of the experimental results, we eliminated abnormal data using Mahalanobis distance. This metric was designed to calculate the distance between points and distributions, which improves the inconsistency and correlation of various dimensional scales in Euclidean distance. The calculation of Mahalanobis distance is not affected by individual dimensions, but it incorporates the internal relationship between various characteristics of the sample data, allowing effective assessment of the data similarity and identification of data anomalies. Sun et al. (2016) combined Mahalanobis distance with Monte Carlo cross‐validation to successfully eliminate outliers in hyperspectral data about tobacco leaf water content. In our study, the adoption of Mahalanobis led to the removal of 15 samples, all from stale beef. We retained 195 valid data samples: 105 from fresh beef and 90 from stale beef.

**FIGURE 4:**
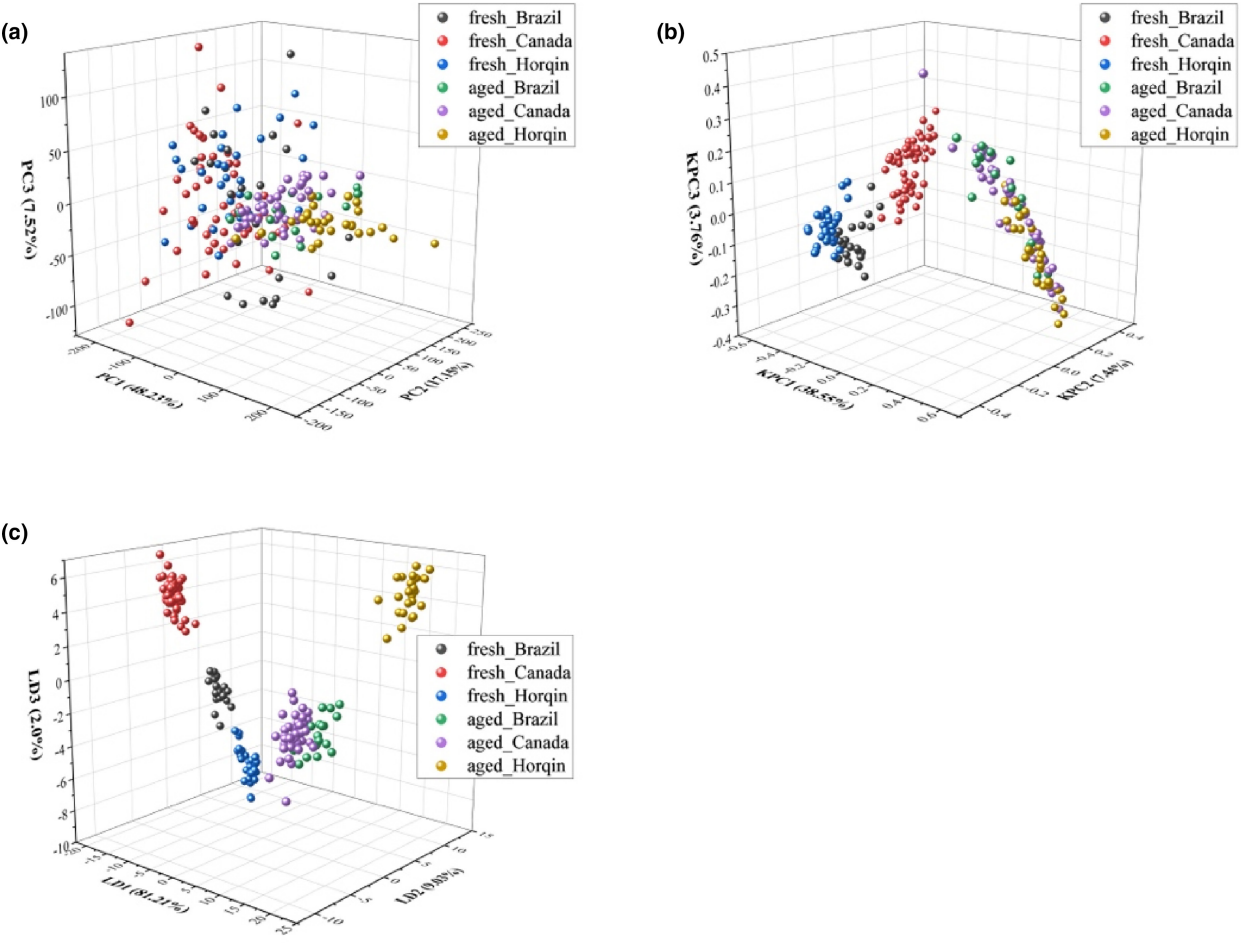
Analysis and comparison of dimensionality reduction results obtained using PCA (a), KPCA (b), and LDA (c).

The characteristic information collected from each sample consists of 22,000 data points. We preprocessed this information to avoid excessive load on our computing resources, and to minimize the contribution from irrelevant information that may affect the performance of pattern recognition models. Principal component analysis (PCA), kernel principal component analysis (KPCA), and linear discriminant analysis (LDA) are commonly used data dimensionality reduction methods in machine learning. The purpose of data dimensionality reduction is to reduce data size and complexity, remove useless information, and increase the recognition accuracy of pattern recognition models by retaining as much characteristic information as possible. We compared the quality and effectiveness of data dimensionality reduction across three different methods: PCA, KPCA, and LDA (see Figure 4). Table 2 details the contribution of each dimension to the original data after dimensionality reduction. Figure 4 shows that LDA achieved significantly better dimensionality reduction than PCA and KPCA. Following LDA dimensionality reduction, fresh and stale beef from different origins appear well separated in feature space: samples of the same class cluster together, while samples from different classes are clearly separated. Table 2 shows that, following PCA and KPCA dimensionality reduction, the first three dimensions contribute <80%, which does not represent the characteristic information of the original data adequately. For comparison, the first three dimensions contribute 92.24% after LDA dimensionality reduction. These results show that LDA can reduce the dimension of the original data while retaining most of the feature information contained in the original data.

**TABLE 2:** Contribution of each dimension for different dimensionality reduction methods.

Method	Contribution rate of no. 1 dimension (%)	Contribution rate of no. 2 dimension (%)	Contribution rate of no. 3 dimension (%)	Total (%)
PCA	48.23	17.15	7.52	72.90
KPCA	38.55	7.44	3.76	49.75
LDA	81.21	9.03	2.0	92.24

### 2.3 Machine learning methods

Thanks to the rapid development of machine learning in recent years, and to its combination with electronic nose technology, electronic noses now play an increasingly important role in several fields. Common pattern recognition models used in conjunction with electronic noses include extreme gradient boosting (XGBoost), logistic regression (LR), K‐nearest neighbor method (KNN), random forest (RF), support vector machines (SVM), and back propagation neural network (BPNN).

#### 2.3.1 Extreme gradient boosting

XGBoost is based on the gradient boosting tree. Its loss function with second derivative may lead to more accurate results, and the regularization term can avoid overfitting. Chen et al. (2021) combined an electronic nose with XGBoost to predict the developmental stage of lung cancer with a prediction accuracy of more than 80%.

#### 2.3.2 Logistic regression

LR is a common classification model in machine learning. It can be used for binary classification and multi‐classification tasks. LR does not assume a specific data distribution, avoiding potential problems caused by inaccurate distributional assumptions. Cano Marchal et al. (2021) combined an electronic nose with LR to predict the quality of virgin olive oil. They obtained an average error of 0.5 unit, with recognition accuracy of 88%.

#### 2.3.3 K‐nearest neighbors

Although KNN is a supervised machine learning method, it does not involve an explicit training process. KNN finds the input K samples nearest to the sample of the training dataset via direct calculation, and identifies a class that includes most of the K samples in the output. Mohamad et al. (2022) combined an electronic nose with KNN to identify two kinds of agarwood: A. Malaccensis and A. Crassna. They obtained accuracy values as high as 94.5%.

#### 2.3.4 Random forest

RF contains multiple decision trees, and its output results are jointly determined by the multiple decision trees it contains. RF has the advantages of high stability, short time consumption, and high precision. Tian et al. (2020) combined an electronic nose with RF to rapidly and successfully detect the acceptability of yogurt flavor.

#### 2.3.5 Support vector machines

SVM is a supervised machine learning algorithm proposed by Cortes and Vapnik. It is widely used in various classification and regression problems. In recent years, many scholars have improved SVM to deal with multi‐classification problems. OVR‐SVM belongs to this category. It is a common multi‐classifier which performs well in multi‐classification problems. Papadopoulou et al. (2011) used an electronic nose to collect characteristic information from beef with different degrees of freshness and then established an SVM recognition model. Their method can successfully distinguish beef samples of different freshness, demonstrating that electronic noses carry great potential for application in the food industry.

#### 2.3.6 Back propagation neural networks

BPNN is an extension of artificial neural network (ANN), and is one of the most widely used neural network algorithms. BPNN consists of forward propagation and back propagation. Forward propagation performs classification or prediction. The main task of backpropagation is to optimize network parameters to minimize the difference between its output and the desired label, so that the network can achieve good classification or prediction. Ren et al. (2018) used an electronic nose equipped with a headspace sampling unit, and combined it with BPNN to evaluate degree of damage in apples. They obtained an accuracy rate of 96.2%.

## 3 RESULTS

We used an electronic nose to collect data from 210 samples of fresh and stale beef. We excluded 15 abnormal samples using Mahalanobis distance (Mahalanobis, 2018), and retained the remaining 195 samples (105 from fresh beef and 90 from stale beef). We used LDA to reduce the dimensionality of the original dataset, and we then applied XGBoost, LR, KNN, RF, SVM, and BPNN recognition models to the reduced dataset with the goal of discriminating between fresh and stale beef.

### 3.1 All pattern recognition models achieved more than 90% accuracy

**FIGURE 5:**
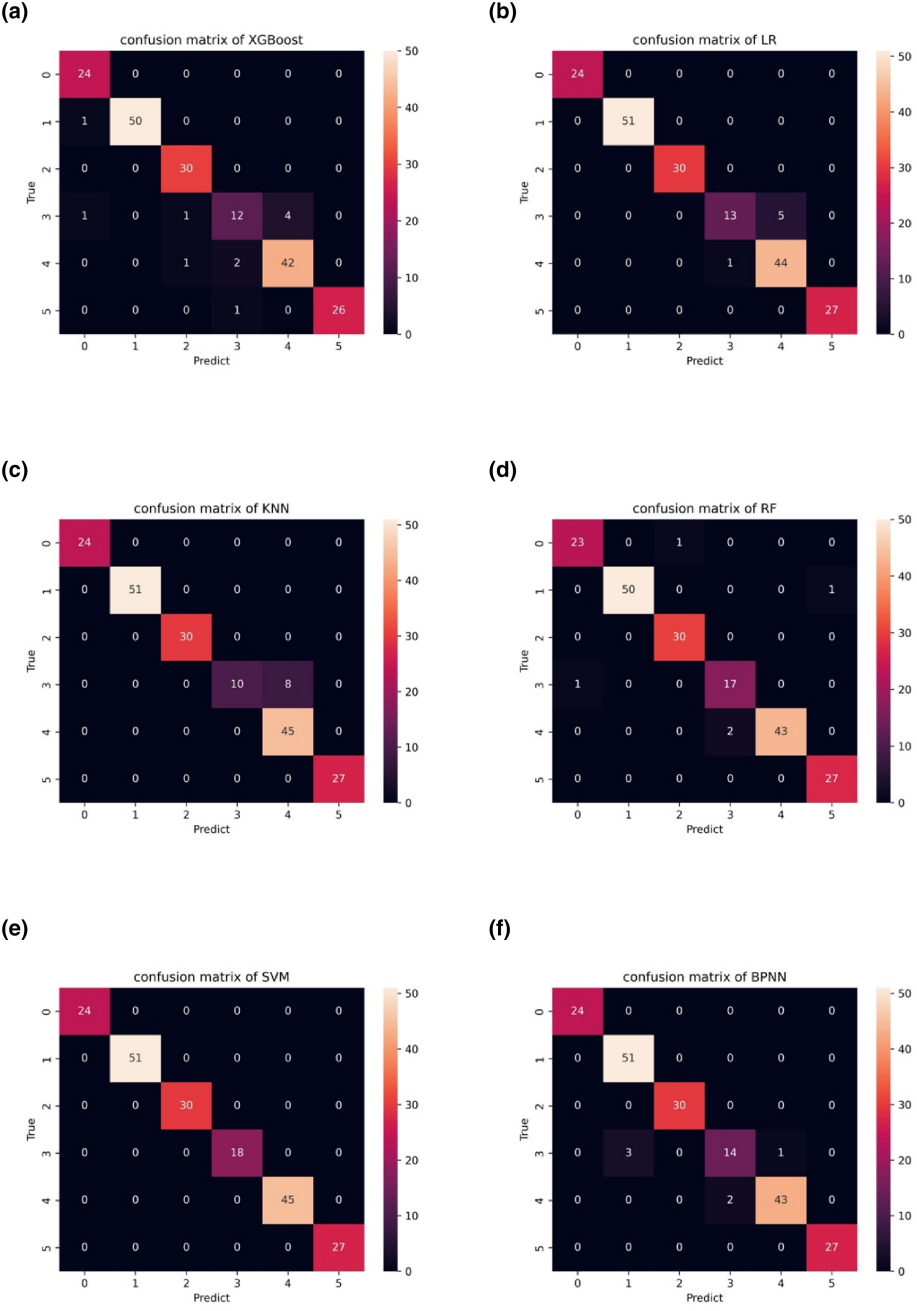
Confusion matrices of XGBoost (a), LR (b), KNN (c), RF (d), SVM (e), and BPNN (f) models.

We used a 10‐fold cross‐validation method to evaluate the performance of each recognition model. The confusion matrix of each model is shown in Figure 5, and model performance is detailed in Table 3. XGBoost, LR, KNN, RF, SVM, and BPNN achieved accuracy values of 94.36%, 96.92%, 95.90%, 97.44%, 100%, and 96.92%, respectively. AUC (Mandrekar, 2010) and *F*1 scores (Goutte & Gaussier, 2005) are crucial metrics for evaluating model performance. We calculated these metrics based on established methods. Their *F*1 scores are 0.943, 0.982, 0.962, 0.974, 1.0, and 0.968, respectively, and the corresponding AUC values are 0.990, 0.989, 0.913, 0.967, 1.0, and 0.992, respectively. The confusion matrix shows that groups 0, 1, and 2 are fresh samples (Brazil, Canada, Horqin), while 3, 4, and 5 are the corresponding aged samples.

**TABLE 3:** Performance values for XGBoost, LR, KNN, RF, SVM, and BPNN recognition models.

Model	Precision	Recall	*F*1 score	AUC	Accuracy (%)
XGBoost	0.942	0.944	0.943	0.990	94.36
LR	0.986	0.979	0.982	0.989	96.92
KNN	0.965	0.959	0.962	0.913	95.90
RF	0.975	0.974	0.974	0.967	97.44
SVM	1.0	1.0	1.0	1.0	100
BPNN	0.968	0.969	0.968	0.992	96.92

### 3.2 SVM achieves satisfactory results

The SVM recognition model achieved perfect accuracy (100%) with *F*1 score and AUC values of 1.0. These results indicate excellent accuracy and stability, demonstrating that electronic nose technology combined with SVM can effectively distinguish between fresh and stale beef on the market. Our approach guarantees the dietary safety of consumers and provides a powerful tool and technical route for the healthy and sustainable development of the beef industry. It opens up a new field for the future combination of electronic nose technology and machine learning in the field of food safety inspection.

## 4 DISCUSSION

In recent years, some businesses have presented stale beef that had been stored for 2 years or more as fresh, and have advertised it for sale on the market to make illegal profits. The nutritional value of stale beef is lost during storage at ultra‐low temperatures. Furthermore, the storage environment of stale beef is often compromised, and sanitation is poor. Stale beef represents a safety hazard for consumers. Our study was motivated by the need to protect their personal health and safety, safeguard their legitimate rights and interests, and to fight the illegal sale of stale beef.

## 5 CONCLUSION

We combined electronic nose technology with machine learning methods (XGBoost, LR, KNN, RF, SVM, and BPNN) to discriminate between stale and fresh beef from different origins, and used a 10‐fold cross‐validation method to evaluate model performance. The results of all the confusion matrix indicate that for fresh samples, the three sources of meat can be effectively distinguished. However, for aged beef, misclassification may occur between meat from Brazil and Canada. This is similar to the results obtained earlier with LDA dimensionality reduction. We have provided different dimensionality reduction methods (PCA, KPCA, LDA), processed by different classification methods (XGBoost, LR, KNN, RF, SVM, BPNN), and provided as many model performance metrics as possible (precision, recall, *F*1 score, AUC, accuracy), thus increasing the credibility and verifiability of the research. All classification methods achieved accuracy values exceeding 95%, and AUC values above 0.91. Our experimental results show that electronic nose technology combined with SVM can carry out this task with perfect recognition accuracy (100%), and that model stability is equally satisfactory. This study lays the foundations for the adoption of electronic nose technology in the field of beef quality assessment. In future research, we hope to predict how long stale beef has been stored for, and whether it is spoiled or not.

## AUTHOR CONTRIBUTIONS

**Wenshen Jia:** Conceptualization (equal); project administration (lead); supervision (lead); writing – original draft (equal). **Haolin Lv:** Data curation (equal); formal analysis (equal); software (equal). **Yang Liu:** Investigation (equal); visualization (equal); writing – original draft (equal). **Wei Zhou:** Funding acquisition (equal); supervision (equal). **Yingdong Qin:** Conceptualization (equal); writing – review and editing (lead).

## CONFLICT OF INTEREST STATEMENT

The authors declare that they have no conflicts of interest.

## Data Availability

Data will be made available from the authors on request.
